# Innovative Technology–Based Interventions to Reduce Stigma Toward People With Mental Illness: Systematic Review and Meta-analysis

**DOI:** 10.2196/35099

**Published:** 2022-05-30

**Authors:** Matías E Rodríguez-Rivas, Adolfo J Cangas, Laura A Cariola, Jorge J Varela, Sara Valdebenito

**Affiliations:** 1 Facultad de Psicología Universidad del Desarrollo Santiago Chile; 2 Department of Psychology, Health Research Centre University of Almería Almería Spain; 3 Department of Clinical and Health Psychology University of Edinburgh Edinburgh United Kingdom; 4 Violence Research Centre, Institute of Criminology University of Cambridge Cambridge United Kingdom

**Keywords:** stigma, mental illness, technology-based, serious games, virtual reality, e-contact, simulation intervention, internet intervention, meta-analysis

## Abstract

**Background:**

Stigma toward people with mental illness presents serious consequences for the impacted individuals, such as social exclusion and increased difficulties in the recovery process. Recently, several interventions have been developed to mitigate public stigma, based on the use of innovative technologies, such as virtual reality and video games.

**Objective:**

This review aims to systematically review, synthesize, measure, and critically discuss experimental studies that measure the effect of technological interventions on stigmatization levels.

**Methods:**

This systematic review and meta-analysis was based on PRISMA (Preferred Reporting Items for Systematic Reviews and Meta-analyses) guidelines and included studies in English and Spanish published between 2016 and 2021. Searches were run in 5 different databases (ie, PubMed, PsycInfo, Scopus, Cochrane Library, and ScienceDirect). Only randomized controlled trials were included. Two independent reviewers determined the eligibility, extracted data, and rated methodological quality of the studies. Meta-analyses were performed using the Comprehensive Meta-Analysis software.

**Results:**

Based on the 1158 articles screened, 72 articles were evaluated as full text, of which 9 were included in the qualitative and quantitative syntheses. A diversity of interventions was observed, including video games, audiovisual simulation of hallucinations, virtual reality, and electronic contact with mental health services users. The meta-analysis (n=1832 participants) demonstrated that these interventions had a consistent medium effect on reducing the level of public stigma (*d*=–0.64; 95% CI 0.31-0.96; *P*<.001).

**Conclusions:**

Innovative interventions involving the use of technologies are an effective tool in stigma reduction, therefore new challenges are proposed and discussed for the demonstration of their adaptability to different contexts and countries, thus leading to their massification.

**Trial Registration:**

PROSPERO International Prospective Register of Systematic Reviews CRD42021261935; https://www.crd.york.ac.uk/prospero/display_record.php?ID=CRD42021261935

## Introduction

### Stigma Toward People With Mental Illnesses

Globally, stigma toward people with mental illness represents a serious public health problem and is considered the main barrier to social inclusion and participation of those impacted. It has a negative effect on their quality of life, worse therapeutic results, and even an increased risk of suicide and mortality [1-3].

Thus, discrimination, prejudice, and stereotypes present in society not only severely impact the recovery process, quality of life, and well-being of people with mental illnesses and their families, but also represent the main gap in accessing specialized mental health services by the general population [4,5]. The aforesaid has great relevance in the global context, where research has shown the presence of high levels of discrimination, stigma, and prejudice toward those impacted by mental health problems, especially schizophrenia and bipolar disorder [6,7].

### Innovative Interventions Carried Out at International and National Levels

Faced with the high levels of stigmatization present in society, several initiatives and studies have been conducted that focused on its reduction. It has been demonstrated that direct contact with people with mental illnesses and educational initiatives are essential and effective interventions to reduce stigma [8,9]. Although direct face-to-face contact with people with mental illness has been shown to be a key component of successful stigma reduction programs, their implementation in virtual learning and innovative spaces is recent [10]. In the last years, several authors have shown that innovative technology-based interventions have had a great impact on the reduction of stigma toward people with mental illness, mainly due to its adaptability to different contexts and age ranges [11]. Among this type of interventions, the use of video games has been highlighted as an effective tool to reduce anxious and depressive symptomatology in patients, and has been useful to reduce misconceptions and stigmatization about severe mental illnesses, such as schizophrenia or bipolar disorder [12,13].

In addition, because of the growth of technologies, the application of virtual and immersive reality in mental health has become increasingly common [14]. For example, it has demonstrated its utility in the treatment of mental health problems, such as phobias and anxiety symptoms, among others [15], along with a reduction of the negative perceptions and attitudes toward people with mental illnesses [16]. This usefulness and effectiveness can be explained by the degree of immersion in a strongly educational environment, which promotes the change of attitudes and beliefs [17]. By contrast, the use of simulation platforms has showed controversial results, with some studies showing an increase of stigmatization when used through the simulation of hallucinatory symptomatology, which can promote negative beliefs and attitudes toward people with mental illness [18,19].

Finally, as an innovative intervention, electronic contact (e-contact)—defined as “computer-mediated real-time interactions where members of different groups interact online” through virtual media with mental health services users [20]—has been used as a strategy for promoting awareness and reducing prejudice among ideologically different groups [21]. However, e-contact implementation in the field of stigma reduction is new and innovative, and it has been demonstrated that e-contact using chat and synchronous videoconferencing can reduce anxiety, stigma, and stereotypes toward the population impacted by mental disorders, and promote an inclusive attitude [11,22].

Despite the considerable progress in this field, further research is needed on innovative technologies and their application in mental health care, such as advances in detection, treatment, and promotion of inclusion and well-being of people with mental health problems [23,24]. Regarding this growing field of application of new technologies and the need to synthesize, measure, and critically discuss the effects of the studies performed for the reduction of stigma, the objectives of this systematic review and meta-analysis are to assess the effectiveness of technology-based interventions to reduce stigma associated with people with mental health problems and to describe the experimental studies that use these types of interventions.

## Methods

### Data Sources and Search Strategy

The systematic review and meta-analysis protocol were registered in the Prospective Register of Systematic Reviews (PROSPERO) international database (registration ID: CRD42021261935) and was conducted according to the guidelines and recommendations of the PRISMA (Preferred Reporting Items for Systematic Reviews and Meta-analyses) [25].

From January to July 2021, searches were conducted from the following 5 databases, including texts dated from March 5, 2016, to March 5, 2021: PubMed, PsycInfo, Scopus, Cochrane Library, and ScienceDirect, using the following string of search terms: [technology or technologies or simulation or virtual or digital or Internet or web or games or computer or app or online or electronic or social media] AND [stigma or discrimination or prejudice or negative attitude or stereotypes] AND [mental health or mental disorder or mental illness or schizophrenia or psychosis or bipolar disorder or depression].

In addition, a final manual trace back literature search was conducted in August 2021 to identify any recently published sources/literature.

### Inclusion and Exclusion Criteria

Published articles that met the following criteria were included: (1) randomized controlled trials using innovative technologies (defined as software apps used with smartphone, videogames, e-contact, or virtual reality); (2) interventions aimed at reducing stigma toward people with mental illness (eg, schizophrenia, psychosis, or bipolar disorder), which included at least one relevant quantitative measure of public stigma (eg, attitudes, stereotypes, and social distance); (3) interventions relevant for any populations (eg, students and general population); (4) all age groups; and (5) articles written in English and Spanish, published in peer-reviewed journals.

Exclusion criteria were (1) reduction of stigma not related to mental health problems; (2) technology using only video or education (eg, films or presentations) not combined with any other innovative technology (eg, virtual reality, videogames, or e-contact); (3) reduction focused on self-stigma only; (4) interventions focused on stigma toward psychiatry or addictions; (5) research protocols; (6) measurement of stigma; and (7) studies that did not include a randomized control group as a comparison.

### Study Selection and Data Extraction

After excluding duplicates using Endnote reference manager software, 2 researchers (MER-R and AJC) independently selected articles for inclusion. A third investigator (LAC) examined all the included articles to review this selection and resolve discrepancies. In addition, to check and ensure consistency and clarity at the screening and coding stages across studies, we calculated the interrater reliability using the Cohen κ coefficient [26]. We obtained a Cohen κ of 0.75 (SE 0.11), demonstrating a medium to high degree of agreement among coders. Following this calculation, coders (MER-R and AJC) reviewed the articles in which they found disagreements, and any discrepancies were resolved by a consensus discussion with a third investigator (LAC) who was not involved in the searches.

The eligibility of search results was examined in 2 stages: first by title and abstract, and then by full text. Reasons for exclusion were recorded for each document excluded.

Data were extracted using a standardized table format, which was then reviewed by a third author (LAC). Characteristics of each article included the study design, sample size, setting, type of new technology applied, control or comparison intervention, as well as the main outcomes and effect sizes of the interventions. In the cases where the data for the calculation of the effect size were incomplete, we contacted the principal investigator to request the necessary additional information.

### Quality and Risk-of-Bias Assessment

To assess the quality of the selected articles, 2 researchers (MER-R and AJC) independently assessed the risk of bias (ROB) of each study using the Cochrane ROB-2 assessment tool, which assesses 7 study design quality criteria (ie, random sequence generation, allocation concealment, blinding of participant and personnel, blinding of outcome assessment, incomplete outcome data, selective reporting, and other bias), graded as high, medium, or low risk [27]. Discrepancies were resolved by further discussions and consensus among the authors. Figure 2 summarizes the assessment of the ROB, which was performed using the robvis visualization tool.

The use of the funnel plot for the evaluation of publication bias was not incorporated, because it has been demonstrated that its use is not reliable when the number of studies pooled in direct comparison is less than 10 [27].

### Statistical Analysis

The meta-analysis was conducted by researchers (SV and MER-R) using the Comprehensive Meta-analysis software (version 2) [28]. Standardized mean difference and the inverse variance method with a 95% CI were used for continuous and normally distributed data, respectively. The *I*^2^ and Q-statistic were used to explore heterogeneity of effect sizes [29]. Random effects models were used due to the heterogeneity in the type of intervention in the studies [30].

As 3 of the studies included more than 1 scale that assessed levels of stigma [11,22,31], we conducted an analysis that combined them into a single effect size. In those cases, we followed the methodology suggestion for complex data structures [30]. As a consequence, we computed a summary effect including the multiple measures; this synthetic effect size was then included in the meta-analysis.

In addition, as 2 of the studies [32,33] reported their results through standardized regression, the β coefficients were entered into the comprehensive meta-analysis software (CMA) as correlation coefficient, according to the recommendations of Peterson and Brown [34].

In addition, we used statistical procedures to quantify the effect of publication bias, by Duval and Tweedie’s trim-and-fill analysis and Rosenthal’s fail-safe N test [35].

## Results

### Output of Searches

**Figure 1:**
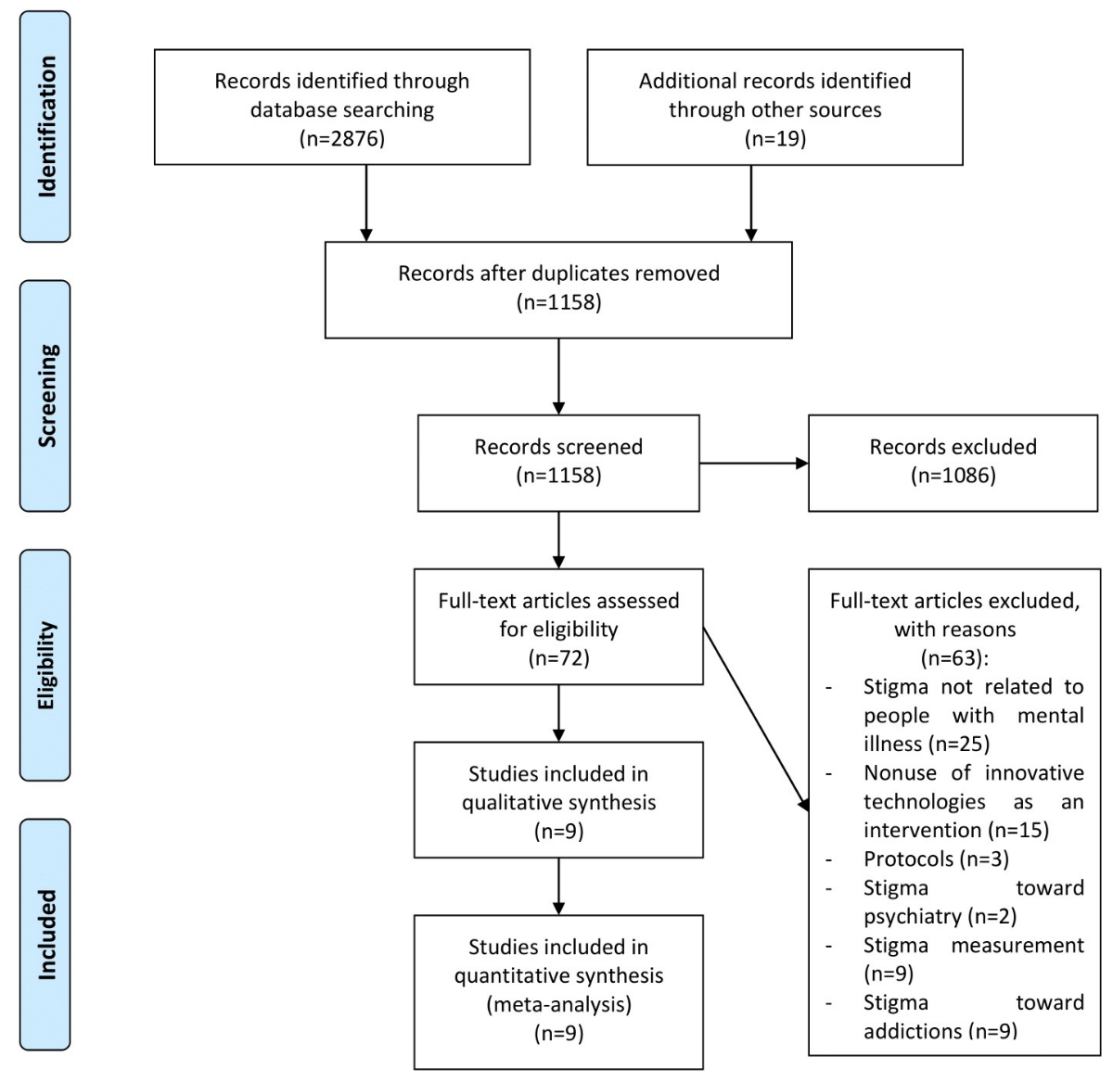
Flowchart of the systematic review process.

Of the 2876 studies retrieved from the selected databases, 1718 duplicates were removed and 1158 were screened. Upon the screening of the titles and abstracts, 1086 studies were removed. For the remaining 72 studies, their full texts were checked, among which an additional 63 were removed due to specific reasons (Figure 1). Only 9 articles presented enough statistical data for meta-analysis.

### Characteristic of Included Studies

The characteristics of the included studies are presented in Table 1. A total of 9 randomized control trial studies were included, which utilized a variety of technology-based interventions to reduce stigma, including interventions using video games (n=4), audiovisual simulation of hallucinations (n=1), virtual reality (n=2), and the use of e-contact with mental health services users through videoconferencing and online chats (n=2). Most of these studies were conducted with undergraduate students (8/9, 89%). As many as 4 studies (44%) were conducted in Europe, 2 (22%) in North America, and only 1 each in Asia (11%), Australia (11%), and Latin America (11%). Most of the participants were female. The proportion of female participants ranged from 50.3% to 81.1%, and the mean age of the participants ranged from 15.7 to 24 years. Public stigma in the studies was measured through different scales, with the most commonly used being the Attribution Questionnaire and the Questionnaire on Student Attitudes Toward Schizophrenia.

**Table 1:** Characteristics of selected studies (n=9).

Experimental intervention	Control group	Sample size	Population	Mean age (SD)	Female, %	Public stigma scale	Outcome public stigma scale (EG^a^ and CG^b^)	Study
e-Contact with a person diagnosed with schizophrenia through an online chat.	Group without intervention.	133	UGS^c^	18.8 (1.5)	64.7	AQ-ER^d^	EG: Decreasing stigma in the factors Fear (*d*=–0.87; *P*<.001) and Anger (*d*=–0.65; *P*<.001) compared with the control condition. Pity did not have a significant difference compared with the CG (*d*=–0.25; *P*=.229).	[22]
						SS-8^e^	EG: Decreasing stigma in the factor Stereotypes (*d*=–0.70; *P*<.001) compared with the CG.	
						PDD^f^	EG: No statistically significant difference in the social distance among EG and CG (*d*=–0.34; *P*>.05).	
e-Contact with a person diagnosed with schizophrenia by videoconferencing.	Cardiovascular health-related activity.	40	UGS	20.6 (1.3)	80.0	AQ-E^g^	EG: Decreasing total stigma level (*d*=–2.33; *P*<.001), and the factors Dangerous-Fear (*d*=–1.73; *P*<.001), Avoidance (*d*=–2.32; *P*<.001), Coercion (*d*=–1.77; *P*<.001), and Lack of Solidarity (*d*=–0.83; *P*=.002). No statistically significant difference in the CG (*P*=.38).	[11]
						QSAS^h^	EG: Decreasing total stigma level (*d*=–1.11; *P*=.004) and the factors Dangerousness (*d*=–1.02; *P*=.007) and Stereotypes (*d*=–.83; *P*=.02). No statistically significant differences among the CG (*P*=.66).	
Audiovisual simulation of hallucination symptoms.	Group without intervention.	244	UGS	18.62 (1.0)	62.7	PDD	EG: No immediate significant change and 1 week later was documented for any of 2 stigma factors evaluated (*P*>.05). No significant differences in the pre-post intervention and 1 week later among the CG (*P*>.05).	[31]
A serious videogame called Stigma-Stop.	A video game unrelated to mental health.	552	HSS^i^	15.78 (2.65)	50.0	QSAS	EG: Decreasing total stigma level (*d*=–0.39; *P*<.001), and the factors Dangerousness (*d*=–0.66; *P*<.001) and Stereotypes (*d*=–1.36; *P*=.001). No statistically significant differences among the CG (*P*=.44).	[36]
A serious videogame called Stigma-Stop.	A video game unrelated to mental health.	530	UGS and HSS	18.51 (4.34)	61.5	QSAS	EG: University students had decreasing total stigma level (*d*=–0.37; *P*<.001) and the factor Social Distance (*d*=–0.65; *P*<.001), but not the factor Stereotypes (*P*=.64).	[37]
							EG: High-school students had reductions in total stigma level (*d*=–0.50; *P*<.001), and the factors Social Distance (*d*=–0.72; *P*<.001) and Stereotypes (*d*=–0.22; *P*<.001). No statistically significant difference among the CG (*P*=.95).	
A videogame that uses avatars with mental illnesses.	Group watched gameplay footage.	207	UGS	20.42 (not reported)	66.7	SDS-6^j^	EG: Structural equation model analysis for both measures show a decrease in Social Distance (B=–0.21; *P*<.05) in the participants that played the videogame compared with the CG.	[32]
						MIS-9^k^	EG: The structural model did not show a significance difference in the reduction of Stereotypes (B=–.09; *P*>.05) in participants that played the videogames compared with the CG.	
A serious videogame called Stigma-Stop.	Group attendance at routine class.	118	UGS	21.17 (5.8)	81.1	AQ-E	EG: Videogame intervention had a significant effect on decreasing the level of Anger (*d*=–0.95; *P*<.001), Dangerousness (*d*=–1.01; *P*<.05), Fear (*d*=–0.94; *P*<.001), Segregation (*d*=–0.87; *P*<.05), Coercion (*d*=–0.39; *P*<.05), and Avoidance (*d*=–1.03; *P*<.05), and increased the level of Help (*d*=–0.54; *P*<.05). The CG had no statistically significant differences for any of the factors (*P*>.05), except for Anger (*d*=–0.11; *P*<.05).	[13]
Virtual reality implemented by video recording.	Group without intervention.	114	UGS	24.0 (6.6)	58	SPS-6^l^	Structural equation modeling analyses that included all participants who positively evaluated the protagonist of the video game showed significantly increased social proximity (B=0.41; *P*=.002).	[33]
Virtual reality by immersive animated story.	Virtual reality exoplanet video.	206	UGS	21.76 (5.04)	55.3	PSA-21^m^	EG: Decreased total stigma for the virtual reality immersive intervention (*d*=–0.44; *P*=.003) and even for 1-week follow-up (*d*=–0.32; *P*=.02) compared with the CG. In the mediation model in the virtual reality immersive intervention a decrease in the level of stigma was reported (B=–0.42; *P*<.001).	[16]

^a^EG: experimental group.

^b^CG: control group.

^c^UGS: undergraduate student.

^d^AQ-ER: Attribution Questionnaire, Emotional Response factors.

^e^SS-8: 8-item Stigmatization Scale.

^f^PDD: Perceived Devaluation and Discrimination Scale.

^g^AQ-E: Attribution Questionnaire, Spanish version.

^h^QSAS: Questionnaire on Student Attitudes toward Schizophrenia.

^i^HSS: high-school student.

^j^SDS-6: 6-item Social Distance Scale.

^k^MIS-9: 9-item Mental Illness Stereotypes.

^l^SPS-6: Social Proximity to persons with schizophrenia Scale.

^m^PSA-21: 21-item Public Stigma and Acceptance Scale.

### Risk of Bias of the Included Studies

**Figure 2:**
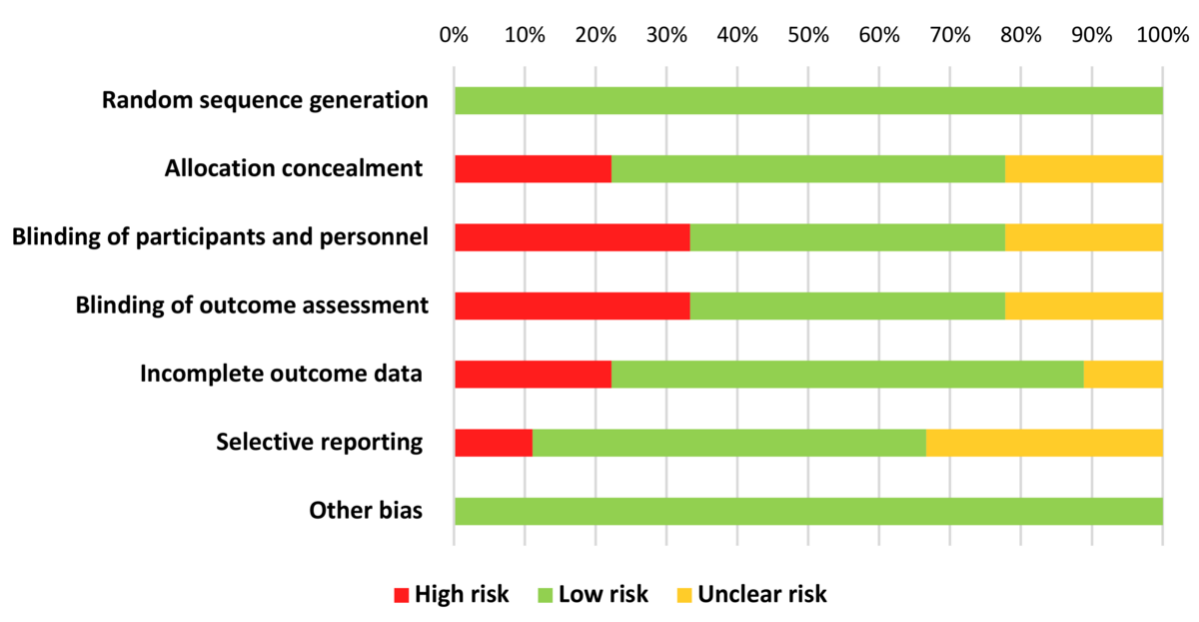
Risk of bias graph: review of authors' judgments about each risk-of-bias item presented as percentages across all included studies.

As shown in Figure 2, most of the studies included in this review were considered as having low ROB in terms of their methodological quality. However, 3 studies [31,32,37] showed a high ROB in the blinding of the participants and blinding of outcome assessment. Besides, 2 other studies [11,32] showed a high ROB related to the allocation concealment. Regarding data, 2 studies [22,37] presented incomplete data. Finally, only 1 study [31] had a high ROB in selective reporting, and no study showed a high risk of other bias or random sequence generation.

### Study and Quantitative Synthesis Outcomes: Public Stigma

**Figure 3:**
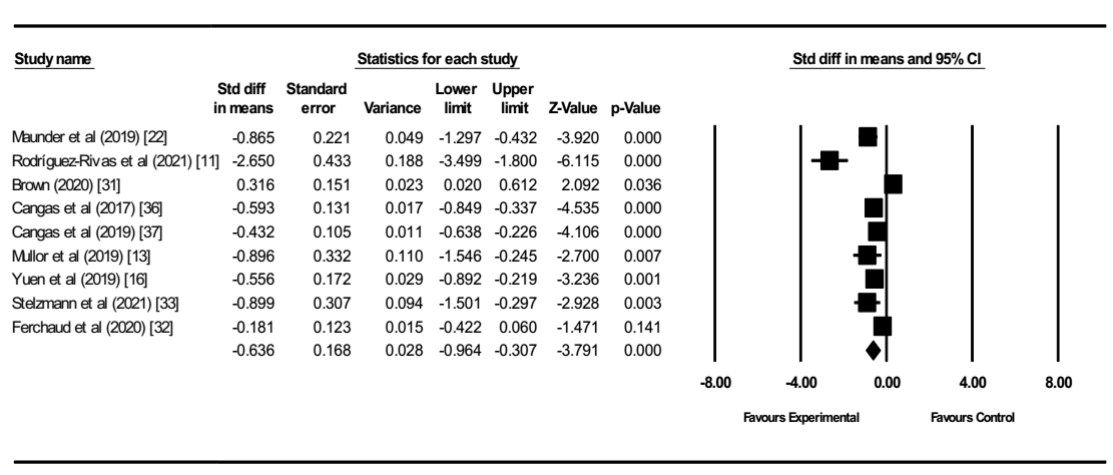
Forest plot comparison of the effect on public stigma (standardized means difference) for the innovation-based intervention and control groups.

A total of 9 articles were included in the meta-analysis, with a total sample of 1832 participants. As shown in Figure 3, the technology-based interventions had medium effects on reducing the level of public stigma (*d*=–0.64; 95% CI 0.31–0.96; *P*<.001) compared with the control group. Only 1 study [31] that used audiovisual simulation symptoms showed an increase in the level of stigma (*P*=.036; *d*=0.32), and another that used videogame with avatars [32] did not show any statistical effect in the level of stigma (*P*=.14; *d*=–0.18). High heterogeneity was observed among the included studies (*P*<.001; *I*^2^=87.6%; *Q*=64.96), which was expected due to the variety of interventions.

### Publication Bias Analysis

We ran statistical analyses for publication bias [35], where Duval and Tweedie’s trim-and-fill analysis identified the differences in effect sizes that could potentially be attributed to bias; the technique imputes effect sizes until the error distribution gets close to normality. In this way, the test offers the best estimate of the unbiased effect [30]. Results suggest that there were no differences in effect sizes attributable to bias. Under a random effect model, the point estimate for the combined studies did not differ when comparing the original and the adjusted estimate (in both cases, standardized mean difference 0.63; 95% CI 0.94-0.30). Based on the parameter of Duval and Tweedie’s trim-and-fill analysis, it seems that no studies are missing.

Additionally, Rosenthal’s fail-safe N test is a technique for computing the number of missing studies that would be necessary to nullify the found effect [38]. Small numbers of missing studies would reveal the likelihood of biased effects. Test was equal to 180, suggesting that it would be necessary to allocate and include 180 missing studies with no effects for every observed study to achieve the combined 2-tailed *P* value exceeding .05. Therefore, it is highly unlikely that missing studies could alter the substantive conclusion.

## Discussion

### Principal Findings

The results of this meta-analysis support the use of new and innovative technology–based interventions to reduce stigma toward people with mental illness.

This study shows a medium effect on stigma reduction, demonstrating a positive impact and effectiveness of these interventions. Its findings are better compared with a previous meta-analysis [39], which reported only a small effect of contact interventions for people with mental illness, and for the educative intervention, both classical and common-type interventions. This is probably due to the increasing technological development, where realism, immersion, and technological interaction are greater, becoming an increasingly natural means of communication and daily application [40].

In this regard, a recent meta-analysis showed that antistigma interventions involving contact had an immediate small-to-medium effect, and it was equivalent with diverse types of contact mediums, such as videos and presentation [41].

Another finding of our study is that the intervention that used simulation of symptoms of hallucination [31] had an increase in the level of stigma, which is concordant with a previous meta-analysis that demonstrated that it can increase social distance and negative attitudes [42]. These negative results may be explained by the focus on symptoms rather than on the recovery process, which may increase stereotypes and prejudice, especially toward people with schizophrenia. Thus, it has been suggested that it should be used with caution and ideally in combination with educational or contact interventions [43]. In this sense, it is important to consider that several research studies show that the information provided is essential to reduce stigma, where, for example, it has been helpful to refer to biographical aspects (eg, related to difficulties, personal adversities), because it allows people to empathize, understand, and generate a change in their attitudes, knowledge, and stigmatizing behaviors [44]. Therefore, the aim is not only to show “symptoms,” but also to promote an understanding of these experiences and the social consequences for the people who experience them [37].

As an additional result, one of the included studies, which used videogames through avatar identification [32], despite showing no significant effect on stigma in the combined effect analysis, reported a significance effect (*B*=–0.21; *P*<.05) for the reduction of social distance, making it a tool that can be considered for future research.

Our study demonstrated the usefulness of innovative interventions in stigma reduction and summarized its latest advances, in accordance with the growing interest and need for the application of new technologies in the field of mental health in the contemporary world. These types of interventions have a variety of advantages and offer innovative solutions to everyday problems, due to their adaptability to different contexts and lower associated cost, along with the possibility of privacy in a protected environment, which allows strategies to be focused and adapted to different key population.

Furthermore, it is important to mention that while stigma is currently one of the most important problems in mental health, there are also a number of socioeconomic, cultural, and structural barriers present in society that limit access to treatment and should be considered as an integral part of reducing discrimination and inequalities in mental health.

### Limitations

This study has some limitations. First, we did not include gray literature, which can increase the risk of publication bias [45]. Despite this, our statistical analysis of publication bias seems to show no missing studies, and that it is also very unlikely that a possible missing study could alter our results. Second, the small number of studies and high heterogeneity prevented us from estimating which technology-based intervention was the most effective, a limitation that may be considered in future studies as the number of publications in this emerging area increases, allowing future subgroup analyses of each type of intervention and addressing the high heterogeneity. Third, all the studies found and included focused on the population of high-school and university students, which may limit the generalizability of the results to the general population. In addition, most of the included studies did not include follow-up studies, which prevented the analysis of long-term stigma reduction.

### Future Research

As stigma is a complex social phenomenon transversally present in the society [1-6], innovative interventions involving use of technologies can be an effective tool for its reduction. However, one challenge in the implementation of this type of intervention is its adaptability to different contexts and countries, and so future interventions should include cross-cultural comparison. In addition, all experimental studies involving technology-based interventions in stigma reduction focus on the young population, which represents a major challenge for future studies focused on the development, implementation, and evaluation of these types of interventions for different ages, such as adults and the elderly [46,47].

Because of the extensive evidence supporting key strategies in the development of antistigma programs, such as the educative and contact components, future research should consider and adapt them to different innovation-based interventions. Its adaptation had demonstrated a great utility, for example, in e-contact with people with mental illness, as they offer the possibility of wider dissemination and even the possibility of reaching remote areas [11,22]. New lines of work should generate greater access and development of low-cost tools with the use of new technologies that allow their use and integration in workplaces, health systems, and educational communities as a daily support tool in mental health, for example, through the development and use of free apps for smartphones and freeware for computers.

Finally, it is essential to generate integrated technological systems not only to reduce stigma, but also to consider preventive interventions in mental health, together with timely referrals to specialized health services and access to treatment. In this regard, we highlight the recently developed app Help Club [48], which provides the possibility of access to communities of mutual support in mental health in virtual spaces, through the use of virtual reality, demonstrating the potential and growing impact of metaverse as a space for social interaction and an increasingly used tool.

### Conclusions

Our meta-analysis showed that innovative interventions involving the use of virtual reality and communication technologies are effective tools for stigma reduction toward people with mental illness and can be an alternative and a complement for the traditional methods on stigma reduction. As this field is growing and emerging, future studies present several challenges in their adaptation and dissemination in different populations and countries.

## Abbreviations

AQ-E: Attribution Questionnaire, Spanish version
AQ-ER: Attribution Questionnaire, Emotional Response factors
CG: control group
e-contact: electronic contact
EG: experimental group
HSS: high-school student
MIS-9: 9-item Mental Illness Stereotypes
PDD: Perceived Devaluation and Discrimination Scale
PRISMA: Preferred Reporting Items for Systematic Reviews and Meta-analyses
PROSPERO: Prospective Register of Systematic Reviews
PSA-21: 21-item Public Stigma and Acceptance Scale
QSAS: Questionnaire on Student Attitudes toward Schizophrenia
ROB: risk of bias
SDS-6: 6-item Social Distance Scale
SPS-6: 6-item Social Proximity to persons with schizophrenia Scale
SS-8: 8-item Stigmatization Scale
UGS: undergraduate student
